# A Case of Double Pregnancy:—One Ovum Being Blighted and Discharged as a Mole; the Other Expelled Two Weeks Afterwards

**Published:** 1867-08

**Authors:** Benj. M. Cromwell

**Affiliations:** Albany, Ga.


### ARTICLE III.

*A Case of Double Pregnancy:—One Ovum being blighted and discharged as a Mole; the other expelled two weeks afterwards.* By BENJ. M. CROMWELL, M. D., Albany, Ga.

April 2d.—Called at night to see Mrs. ——, who, the morning previously, after having two loose actions from her bowels, experienced contractile pains in the abdomen, which she attributed to the tenesmus and dysenteric action. A

few hours afterwards, while superintending her gardening, suddenly, without premonition, she experienced another pain, attended with a discharge of blood and water. Knowing herself to be two or three months pregnant, she at once suspected abortion, but failed, on close inspection to discover any evidence of it, except the discharge above mentioned.

More or less hemorrhage continuing until night, I was sent for. I found her restless, suffering with pain and fatigue in the back, and headache. Pulse normal; hemorrhage not serious, and under influence of lead and opium, gradually ceased on the following day. During the time of its (the hemorrhage's) continuance, clots were discharged, but no organized mass.

The next day, the patient continuing to do well, and the hemorrhage subsiding to the usual discharge, my visits were discontinued.

On the 9th, the husband visited me at my office, and said his wife's catamenia had returned, and asked if it was not unusual for it to return so soon after a miscarriage.

Doubting it to be really a return of the catamenia, I questioned him closely as to its nature, amount, and constitutional effect. He said it was of the color of brick dust; was, if anything, a little less than normal, and was attended with no unpleasant constitutional disturbance—that his wife was going about her usual avocations, had a good appetite, and slept well at night. I advised no treatment, but requested to be kept informed as to the case.

On the 10th, I was again sent for. She was having well marked uterine contractions, headache, and pain in the back, as before. Under the influence of the uterine action, large clots were expelled, and the hemorrhage, though not profuse, was sufficient to cause uneasiness to her medical attendant. Believing it useless to attempt to save the ovum, she was given the wine of ergot freely, and cold cloths were placed over the pubes. Under the influence of these measures, uterine contractions became quite severe, and about 10 o'clock at night, the uterus expelled a carnified mass, oval in its general contour, lobular, convex on its entire surface, and concave within; measuring, in its largest diameter, from 2½ to 3 inches. Attached to its inner concave surface was a membranous sack, with what appeared to be the rudimentary end of a funis.

Under the influence of an opiate, she rested well, and, for the two succeeding days, the discharge ceased almost entirely.

Believing now, that the cause of trouble had ceased to operate, I expected her to convalesce, and was, therefore, unprepared to find febrile symptoms supervening. It seemed evident that this mass was a mole—a conception that had perished, perhaps, while yet an embryo, and had become gelatinised and absorbed, while the diseased placenta, receiving nourishment from the uterus, continued to its present dimensions.

I could see no good reason for asking for a vaginal examination. The lochial discharge continued; but pain in and about the region of the uterus, with the usual pain in the back, was also present.

On the 13th, before the usual hour of my visit, I was again sent for. The patient was having well marked uterine contractions, with discharges of clots and blood. On examination per vagina, I discovered protruding from the os, a soft gelatinous substance, which, on further examination by the speculum, I found was a portion of placenta undergoing decomposition. It was so soft that instruments could get no hold on it, so the finger was introduced in and through the os as far as it would go, and, with the nail, as much of the mass was detached as reached. This was repeated several times, and each time a portion was detached. She was then put on the wine of ergot, and in a few hours the remainder was expelled.

Hemorrhage ceased, and under wine, iron, and quinine, the patient gradually regained her usual health.

The appearance of this second placenta, which was healthy in its structure, can only be accounted for by supposing this to have been a case of double pregnancy—one ovum of which was blighted, while the other was in progress of development. The non-appearance of the fœtus of this second placenta is to be regretted, as it tends to obscure the case; but from its early age and small size, it can readily be conceived to have been lost without detection.

Montgomery, in his "Signs and Symptoms of Pregnancy," treats this subject (moler pregnancy) more fully than any author that I am acquainted with. Madame Boivin, also, has an excellent chapter on this subject.

These authors mention several cases coming under their own, and others' observation, illustrating the above case,—cases of double pregnancy, where one of the products of conception becomes blighted, without in any way interfering with the proper development of the other. According to these authors, the mole may be expelled at the second, third, or any intervening month, up to the time of delivery, without causing premature labor. The mole is sometimes expelled with secundines at the time of delivery; and again, instances are recorded where the mole remains in the uterus for an indefinite period after the usual time of utero-gestation. Ambrose Pari mentions the case of a mole remaining in utero seventeen years.

In a medico-legal point of view, the subject of moler pregnancy is one of vast importance, and merits the closest attention of the physician. A hastily expressed opinion of a medical man may do great injury to chastity and virtue, and cast a stain on the reputation of an innocent married woman. Such instances are recorded.

